# Cooled radiofrequency ablation of the genicular nerves for chronic pain due to osteoarthritis of the knee: a cost-effectiveness analysis based on trial data

**DOI:** 10.1186/s12891-019-2681-2

**Published:** 2019-06-26

**Authors:** Mehul Desai, Anthony Bentley, William A. Keck, Thomas Haag, Rod S. Taylor, Helen Dakin

**Affiliations:** 1 International Spine Pain and Performance Center, Washington, DC USA; 2 Mtech Access, Bicester, UK; 3 Avanos Medical, Alpharetta, GA USA; 4 Maelor Hospital, Wrexham, UK; 5 Institute of Health Research, University of Exeter Medical School, Exeter, UK; 6 Nuffield Department of Population Health, University of Oxford, Oxford, UK

**Keywords:** Cost-utility analysis, QALY, Economic analysis, CRFA, Intra-articular steroid, Oxford knee score

## Abstract

**Background:**

For patients with painful knee osteoarthritis, long-term symptomatic relief may improve quality of life. Cooled radiofrequency ablation (CRFA) has demonstrated significant improvements in pain, physical function and health-related quality of life compared with conservative therapy with intra-articular steroid (IAS) injections. This study aimed to establish the cost-effectiveness of CRFA compared with IAS for managing moderate to severe osteoarthritis-related knee pain, from the US Medicare system perspective.

**Methods:**

We conducted a cost-effectiveness analysis utilizing efficacy data (Oxford Knee Scores) from a randomized, crossover trial on CRFA (NCT02343003), which compared CRFA with IAS out to 6 and 12 months, and with IAS patients who subsequently crossed over to receive CRFA after 6 months. Outcomes included health benefits (quality-adjusted life-years [QALYs]), costs and cost-effectiveness (expressed as cost per QALY gained). QALYs were estimated by mapping Oxford Knee Scores to the EQ-5D generic utility measure using a validated algorithm. Secondary analyses explored differences in the settings of care and procedures used in-trial versus real-world clinical practice.

**Results:**

CRFA resulted in an incremental QALY gain of 0.091 at an incremental cost of $1711, equating to a cost of US$18,773 per QALY gained over a 6-month time horizon versus IAS. Over a 12-month time horizon, the incremental QALY gain was 0.229 at the same incremental cost, equating to a cost of US$7462 per QALY gained versus IAS. Real-world cost assumptions resulted in modest increases in the cost per QALY gained to a maximum of US$21,166 and US$8296 at 6 and 12 months, respectively. Sensitivity analyses demonstrated that findings were robust to variations in efficacy and cost parameters.

**Conclusions:**

CRFA is a highly cost-effective treatment option for patients with osteoarthritis-related knee pain, compared with the US$100,000/QALY threshold typically used in the US.

**Electronic supplementary material:**

The online version of this article (10.1186/s12891-019-2681-2) contains supplementary material, which is available to authorized users.

## Background

Symptomatic knee osteoarthritis is a non-inflammatory degenerative disease that can cause substantial pain and negatively impact patients’ usual activities, affecting more than 9 million adults in the US [1]. In addition to reducing health-related quality of life, knee osteoarthritis is a major economic burden with surgical treatment options alone estimated to cost around US$12 billion a year [2] and the societal impact of absenteeism resulting from osteoarthritis estimated at a similar value [3].

Arthroplasty is an effective and established terminal therapeutic option for late-stage osteoarthritis-related pain and dysfunction [4, 5]; however, the procedure may not be appropriate in all patients due to co-morbidities, lack of social support, or other factors [6, 7]. Intra-articular steroid (IAS) injections are often considered a first-line invasive treatment and can provide short-term pain relief, but may require repeated treatment, and may cause cartilage damage when used over an extended period [8, 9].

Outpatient-delivered radiofrequency ablation of targeted genicular nerves has emerged as a realistic, minimally-invasive procedural option for patients with pain associated with knee osteoarthritis [10–12]. In particular, Coolief™ System (Avanos Medical, Alpharetta, GA, USA), the cooled form of radiofrequency ablation (CRFA), is an effective and safe long-term therapeutic option for managing pain and improving physical function and health-related quality of life in patients with knee osteoarthritis [13–17] and to date is the only radiofrequency treatment to be approved by the FDA for management of osteoarthritis knee pain.^1^

In a randomized, controlled, open-label, multicenter, crossover trial (NCT02343003), CRFA reduced knee pain (measured by numeric rating scale) by at least 50% in 74% of subjects compared with 16% of subjects treated with IAS injection (*p* < 0.0001) at 6 months’ follow up [14], with improvements in knee pain in the CRFA arm sustained out to 12 months [13]. Similarly, CRFA resulted in significant improvements in Oxford Knee Score (OKS) at both 6 and 12 months post-treatment [13, 14]. Moreover, subjects originally randomized to IAS who were then treated with CRFA (crossover) at 6 months (*n* = 58), after being dissatisfied with their IAS treatment, subsequently achieved statistically-significant and clinically-relevant pain relief and functional improvements [13].

Although the comparative clinical effectiveness and safety of CRFA has been clearly demonstrated, no studies have yet assessed the cost-effectiveness of this procedure. Cost-effectiveness analysis is a standard approach for estimating the resources used (costs) and the health benefits achieved for a medical intervention compared with an alternative strategy.

The current study aimed to evaluate the cost-effectiveness of CRFA compared with IAS injection for moderate to severe pain due to knee osteoarthritis from the US Medicare system perspective, by way of a decision analysis model utilizing outcomes from clinical trial NCT02343003 and costs from routine practice.

## Methods

### Economic analysis overview

The cost-effectiveness analysis was developed in Microsoft Excel to evaluate the costs and health outcomes of patients undergoing CRFA or IAS. The analysis was based on the pivotal clinical trial NCT02343003 [13, 14], and mirrored the trial in terms of the interventions compared, the time-horizon considered, the procedures performed and the settings of care in which patients were managed. Comparative efficacy data (mean OKS) from the trial were used to determine health gains achieved by patients undergoing each therapy in terms of quality-adjusted life-years (QALYs). Costs were estimated from the US Medicare perspective. The primary outcome was the cost per QALY gained, which captures both the health gains and healthcare costs associated with treatment. We calculated this incremental cost-effectiveness ratio (ICER) as the difference in total cost between CRFA and IAS injection, divided by the difference in QALYs. ICER values were calculated where CRFA resulted in health benefits (increased QALYs) at an increased total cost.

### Economic analysis design

Clinical study NCT02343003 was a prospective, randomized, open-label, multicenter crossover trial and has been reported in detail by Davis et al. [13, 14]. The population considered in the economic analysis reflected the population enrolled in the trial, being patients with radiographic confirmation of grade 2 to 4 knee osteoarthritis within 12 months prior to study screening and knee pain for 6 months or more that was unresponsive to conservative treatments. Other inclusion criteria included: numeric rating scale pain score of 6 or greater for the index knee; OKS of 35 or less; positive diagnostic genicular nerve block, defined as a decrease of ≥50% in numeric rating scale score; if taking an opioid or other morphine-equivalent medication, the dose must have been clinically stable.

In the trial, subjects were randomized 1:1 to receive CRFA (*N* = 76) utilizing the Coolief™ System (Avanos Medical, Alpharetta, GA, USA) or a single IAS injection with corticosteroid (*N* = 75), and were assessed at study baseline and at 1, 3, 6 and 12 months post-intervention [13, 14]. The ablation technique has been described in detail previously [13, 14]. The crossover design allowed any subject who was dissatisfied with their IAS treatment after their 6-month visit, which may have included an increase in knee pain, to choose to cross over and receive CRFA treatment [13]. Crossover subjects were assessed at 7, 9 and 12 months post-study baseline (corresponding with 1, 3 and 6 months post CRFA treatment).

Our primary economic analysis compared CRFA with IAS, using time-horizons of either 6 or 12 months post-treatment, consistent with the interventions and follow up periods within the trial. The analysis did not extend beyond this timeframe as to do so would have required assumptions to be made as to the durability of the treatment effect and the need for re-treatment beyond this timeframe, and introduced uncertainty into the analysis. In line with the trial, our primary analysis assumed that patients received one intervention with either CRFA or IAS at study baseline and the benefits and costs included in the analysis reflect those associated with this baseline intervention. No repeat treatments were included. In a secondary analysis (12-month crossover scenario), the CRFA group was also compared with the crossover group out to 12 months; in this analysis, all patients in the crossover group received IAS at baseline, followed by CRFA at 6 months. Costs and benefits were not discounted because the time horizon was only 6–12 months.

All patients screened for the trial underwent diagnostic genicular nerve block to determine their potential to respond to CRFA, and thus determine their eligibility for trial inclusion; 233 patients were screened, of which 151 met the inclusion criteria for the trial, with six failing to achieve a positive genicular nerve block response (3.8%). Due to the high costs associated with a genicular nerve block (Table 3) we have included this screening test in our cost analysis. In clinical practice, this would only need to be performed for patients who were going to receive CRFA and not those treated with IAS, and therefore we have conservatively applied the cost of genicular nerve block to the CRFA arm only. In this CRFA arm, we assumed that 96.2% of subjects (those who had a positive response to the nerve block) accrued the costs and benefits associated with CRFA, whereas 3.8% who failed to achieve a positive response were assumed to receive the costs and clinical benefits associated with IAS.

### Clinical inputs and health utilities

The economic model calculated health benefits in the form of QALYs by mapping from trial-based changes in knee function, as measured using mean OKS. The OKS is a widely used measure of knee function and pain which was developed and validated to assess patient outcomes following knee replacement [18] and has been employed across trials, cohort studies and audits [19]. The OKS captures patients’ assessment of knee symptoms and function with 12 questions which can be scored from 0 (worst function) through to 4 (best function), with a total possible score ranging between 0 and 48 [14, 19].

To enable the use of OKS in the economic analysis and allow the estimation of health gains in the form of QALYs, OKS data was converted into utility scores using a published mapping algorithm. Utilities range from 1 (perfect health) to zero (death). The EQ-5D is the most widely-used utility measure employed in studies that estimate QALYs [20] and is preferred by health technology assessment bodies, such as the National Institute for Health and Care Excellence and the Canadian Agency for Drugs and Technologies in Health [21, 22]. Mapping has become a well-recognized method to estimate utilities for use in economic analyses, and involves statistically predicting utilities from a condition-specific measure, such as the OKS [23]. We utilized mean OKS, as reported in the clinical trial, and mapped these to EQ-5D utility scores using a validated mapping algorithm estimated by Dakin et al. [24] that maps to utilities using the UK time trade off EQ-5D (3 level) tariff [25]. Although Dakin et al. also estimated a response mapping with better prediction accuracy, we used a simple model reported in the same paper which allowed us to use mean OKS without patient-level data.

$$ Predicted\  EQ-5D\  utility=-0.0404485+0.0224412\ast mean\  OKS $$

Where mean OKS = mean OKS value from trial population.

Standard errors around mapped utilities were estimated using the spreadsheet tool accompanying the OKS algorithm [24] using methods described by Lawrence et al. [26]. For the primary 6- and 12-month comparisons of CRFA with IAS, we utilized mean OKS results from the full analysis set as per the clinical trial primary analysis [13, 14] (Table 1); 58 and 67 subjects had OKS data available for analysis in the CRFA and IAS arms, respectively, at the 6-month follow up [14]. Fifty-two subjects in the CRFA arm and 3 subjects in the IAS arm completed the study in their original arm and had OKS data at the 12-month follow-up [13].

**Table 1:** OKS and predicted utility scores (EQ-5D)^a^: used to model CRFA and IAS in primary analysis

		Baseline^b^		Month 1		Month 3		Month 6		Month 12	
		CRFA	IAS	CRFA	IAS	CRFA	IAS	CRFA	IAS	CRFA	IAS
OKS	Mean (SD)	16.7 (4.4)	16.9 (5.1)	33.3 (9.2)	29.4 (8.5)	34.6 (8.3)	24.6 (7.6)	35.7 (8.8)	22.4 (8.5)	34.3 (11.1)	22.0 (16.6)
	Sample size^c^	76	75	67	69	65	68	58	67	52	3
	SE	0.50	0.59	1.13	1.03	1.02	0.93	1.16	1.04	1.54	9.61
Mapped EQ-5D	Mean (SE)	0.335 (0.029)	0.340 (0.030)	0.708 (0.033)	0.619 (0.031)	0.735 (0.032)	0.512 (0.030)	0.761 (0.035)	0.463 (0.032)	0.729 (0.042)	0.453 (0.238)

Abbreviations: *CRFA* cooled radiofrequency ablation, *IAS* intra-articular steroid, *OKS* Oxford Knee Score, *SD* standard deviation, *SE* standard error

^a^Includes all available observations

^b^Time points as per clinical trial NCT02343003

^c^Sample size represents subjects with available data at each time point. Based on full analysis set from clinical trial NCT02343003

For the secondary 12-month crossover scenario, OKS data for the CRFA arm was modelled as per the primary economic analysis. For the crossover arm, we used mean OKS data across all patients randomized to the IAS arm out to 3 months (as per the primary analysis, Table 1), after which time we used mean OKS for the IAS subjects who crossed over to CRFA out to 12 months; post-crossover OKS data were derived from the per-protocol dataset, as per the 12-month clinical trial analysis [13]. Mean OKS and estimated utility scores are provided in Table 1 for CRFA and IAS groups and in Table 2 for the crossover group.

**Table 2:** OKS and predicted utility scores (EQ-5D): used to model the crossover arm^a^

		Study Month 6^b^	Study Month 7^b^	Study Month 9^b^	Study Month 12^b^
OKS	Mean (SD)	18.6 (6.6)	30.0 (9.4)	30.3 (10.0)	29.8 (10.6)
	Sample size^c^	42	40	38	37
	SE	1.02	1.49	1.62	1.74
Mapped EQ-5D	Mean (SE)	0.377 (0.042)	0.633 (0.043)	0.640 (0.046)	0.628 (0.049)

Abbreviations: *CRFA* cooled radiofrequency ablation, *IAS* intra-articular steroid, *OKS* Oxford Knee Score, *SD* standard deviation, *SE* standard error

^a^Based on the clinical trial NCT02343003, subjects in the IAS arm were able to cross over to CRFA at study Month 6 and continued to be followed up at study Month 7, 9 and 12. Data shown for those patients in the IAS arm who received CRFA at month 6

^b^Time points as per clinical trial

^c^Sample size represents only subjects who crossed over from IAS to CRFA at 6 months, with available data at each time point (based on per protocol analysis set)

We assumed that utilities changed linearly between follow-up times and calculated QALYs as the area under the curve.

### Costs

Costs were derived from Centers for Medicare and Medicaid Services fee schedules [27], and included standard physician (in-office or in-hospital) and hospital payments for IAS, CRFA and genicular nerve block procedures. The reference year for costs was 2017. All costs considered in the analysis were assumed to be accrued at the point when patients received their CRFA or IAS intervention. In line with the trial protocol, we assumed that patients would not receive a repeat treatment with CRFA or IAS or arthroplasty during the 6 to 12-month time horizon. Although some subjects in both treatment arms required analgesic medication (opioid and non-opioid) at baseline and throughout the clinical study, there were no significant differences between treatment arms, except for a statistically significant reduction in non-opioid analgesia dose in the CRFA arm at 6 months [13, 14]. Costs of analgesia were therefore conservatively excluded from our analysis. We assumed that following treatment, patients would be discharged to home with instructions for self-care. In practice, patients in both arms may have subsequent nurse follow up and physiotherapy; it was assumed that this pathway of care would be the same for both treatment arms and hence costs for these contacts were excluded from the analysis.

Primary analyses reflected the settings of care and procedures administered in the clinical trial (Summary costs: Table 3; Unit costs: see Additional file 1: Table S1). The cost applied for the CRFA procedure assumed that the procedure was done in a hospital outpatient setting and comprised ablation of the index knee at three anatomic locations, using fluoroscopic visualization of anatomical landmarks for accurate CRF probe placement, consistent with the procedures and settings of care utilized in the trial [13, 14]. Similarly, for the IAS procedure, costs were estimated assuming patients received one corticosteroid injection in the index knee using ultrasound guidance in a hospital outpatient visit. All subjects in the trial underwent genicular nerve block to determine their potential to respond to CRFA, and thus determine their eligibility for trial inclusion; as such this cost was also included in the analysis for all patients in the CRFA arm, assuming an outpatient setting (See “Economic analysis study design”). Patients in the IAS arm were assumed not to receive nerve block, consistent with clinical practice. In the crossover scenario, we assumed that all patients in the crossover arm would receive genicular nerve block.

**Table 3:** Total treatment costs applied in the economic analysis (US$)^a^

	Primary analyses	Real-world cost scenario 1	Real-world cost scenario 2
CRFA procedure	1497.50 (OP)	1497.50 (OP)	1497.50 (OP)
IAS procedure	294.52 (OP)	67.73 (IO)	67.73 (IO)
Genicular nerve block	553.85 (OP)	553.85 (OP)	175.86 (IO)

Abbreviations: *CRFA* cooled radiofrequency ablation, *IAS* intra-articular steroid, *IO* in-office, *OP* outpatient

^a^Based on appropriate Current Procedural Terminology codes, assuming physician and hospital outpatient department payments

Secondary cost analyses (Real-world cost scenarios 1 and 2) were also conducted to account for any differences likely to be encountered in clinical practice. Real-world cost scenario 1 assumed that IAS was administered in the office setting without the need for ultrasound guidance, to better reflect clinical practice for this procedure. Real-world cost scenario 2 assumed that both IAS and genicular nerve block (for CRFA) would be performed in the office setting rather than the hospital outpatient setting; this scenario is likely to be most reflective of real-world settings for CRFA, IAS and nerve block procedures in US clinical practice.

### Data analysis and sensitivity analysis

Conclusions are based on a US$100,000 per QALY threshold based on the current benchmark published by the Institute for Clinical and Economic Review in the US [28]. The intervention is deemed cost-effective if the ICER falls below this threshold and not cost-effective otherwise.

One-way sensitivity analyses were conducted to assess the sensitivity of the model results to changes in efficacy and cost inputs. For all model parameters, except for costs, the minimum and maximum plausible values for one-way analysis were defined as the lower and upper 95% confidence limits. No measure of uncertainty was available for costs and so the plausible range was defined based on ±10% of the mean (See Additional file 1: Table S2). Results of one-way sensitivity analyses were depicted on tornado diagrams, showing how changes in individual model inputs between plausible minimum and maximum values influenced the ICER. We quantified the uncertainty around the conclusions using probabilistic sensitivity analyses, in which all parameters were varied independently except for the utility mapping coefficients, which were correlated [24]. Costs were assumed to follow gamma distributions, assuming that the ±10% plausible range equaled the 95% confidence interval, and clinical inputs were varied using a using a normal distribution defined by their mean and SE. Results of probabilistic sensitivity analyses were depicted on scatter plots on the cost-effectiveness plane, showing the distribution of ICERs generated from 10,000 replicates. In addition, cost-effectiveness acceptability curves depict probabilistic sensitivity analyses results by showing the probability that CRFA would be cost-effective versus IAS, over a range of monetary values that a decision-maker may be willing to pay per QALY.

Subjects in the trial arms were well-matched at baseline, although there was a small, non-significant imbalance in baseline OKS between the CRFA and IAS groups, with the IAS group having a slightly more favorable OKS [14], equating to a slightly higher baseline mean utility score; we adjusted for this difference in a sensitivity analysis, by using the mean baseline OKS for the overall trial population and applying the relative change from baseline for each arm. Sub-group analyses were not presented for other trial endpoints [13, 14], and hence we did not consider any specific patient sub-groups in our economic analyses.

## Results

### Primary analyses

At a time-horizon of 6 months post-treatment, CRFA was associated with a 0.091 gain in QALYs and an incremental cost of US$1711 compared with IAS. CRFA therefore cost US$18,773 per QALY gained (Table 4). Extending the time horizon to 12 months reduced the ICER to US$7462 per QALY, reflecting the sustained benefit of CRFA compared with IAS over 12 months (QALY gain 0.229), while treatment costs are all accrued at the point of treatment.

**Table 4:** Primary analysis, CRFA vs IAS 6- and 12-month time horizon: deterministic mean (probabilistic sensitivity analysis mean; 95% confidence interval)

Time horizon	Intervention	QALYs	Incremental QALY gain	Costs (US$)	Incremental cost (US$)	Cost per QALY gained (US$)^a^
6 months	CRFA	0.347 (0.345; 0.265, 0.420)	0.091 (0.090; −0.024, 0.198)	$2005 ($2005; $1929, $2083)	$1711 ($1711; $1632, $1791)	$18,773 ($19,053; −$58,845, $91,604)
	IAS	0.256 (0.255; 0.174, 0.335)		$295 ($294; $275, $315)		
12-months	CRFA	0.714 (0.706; 0.543, 0.853)	0.229 (0.230; 0.011, 0.440)	$2005 ($2005; $1929, $2083)	$1711 ($1711; $1632, $1791)	$7462 ($7424; $3189, $31,405)
	IAS	0.485 (0.475; 0.323, 0.630)		$295 ($294; $275, $315)		

Abbreviations: *CRFA* cooled radiofrequency ablation, *IAS* intra-articular steroid, *QALY* quality-adjusted life-years

^a^Cost per QALY gained for the comparison of CRFA with IAS

### Secondary analyses

In the 12-month crossover scenario, patients in the IAS crossover group gained the clinical benefit of CRFA from 6 months onwards but accrued the cost of IAS at Month 0 and that of CRFA at Month 6. In this scenario, CRFA was dominant over IAS/CRFA crossover (more effective and less costly), gaining 0.134 QALYs and saving $294.52 compared with IAS/CRFA crossover.

Real-world costing scenarios 1 and 2 generated ICERs that were all below $22,000 per QALY (Table 5). Scenario 1 represents the most conservative cost analysis (IAS in-office not outpatient, nerve block only applied to CRFA patients and still performed in outpatient setting), whereas scenario 2 is likely most representative of the real-world setting (IAS in-office not outpatient, nerve block only applied to CRFA patients but performed in-office).

**Table 5:** Secondary analyses: deterministic mean differences between CRFA versus IAS

Analysis	Incremental QALY gain	Incremental cost (US$)	Cost per QALY gained (US$)
Real world cost scenario 1: IAS in-office, CRFA outpatient, nerve block only applied to CRFA group and still performed in outpatient setting			
6-month time horizon	0.091	1929.97	21,166
12-month time horizon	0.229	1929.97	8296
Real world cost scenario 2: IAS in-office, CRFA outpatient, nerve block only applied to CRFA patients but performed in-office			
6-month time horizon	0.091	1550.98	17,564
12-month time horizon	0.229	1550.98	6768

Abbreviations: *CRFA* cooled radiofrequency ablation, *IAS* intra-articular steroid, *QALY* quality-adjusted life-years

### One-way sensitivity analyses

Adjusting for the small differences in baseline utility between CRFA and IAS groups had a minimal impact on results, reducing the ICER to $17,827 per QALY at 6 months and $7308 per QALY at 12 months.

**Fig. 1:**
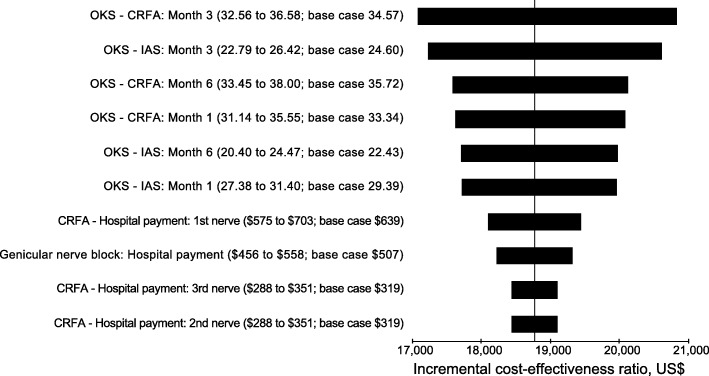
One-way sensitivity analysis, CRFA vs IAS (6-month time horizon). Abbreviations: *CRFA* cooled radiofrequency ablation, *IAS* intra-articular steroid, *OKS* Oxford Knee Score. Vertical line denotes incremental cost-effectiveness ratio for primary analysis

One-way sensitivity analyses demonstrated that the ICER was relatively insensitive to variations in key parameters (Fig. 1). The top 10 parameters generating the most variation in the ICER were changes in OKS (CRFA and IAS at 1, 3 and 6 months) and hospital procedure payments for CRFA (1st, 2nd, 3rd nerve) and IAS.

### Probabilistic sensitivity analysis

**Fig. 2:**
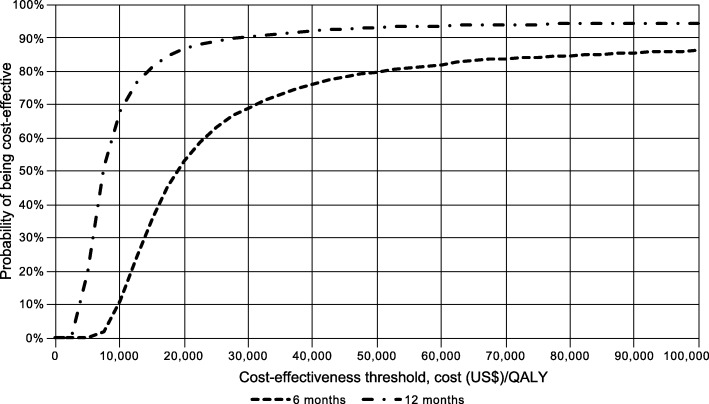
Cost-effectiveness acceptability curves CRFA vs IAS (6- and 12-month time horizons). Abbreviations: *CRFA* cooled radiofrequency ablation, *IAS* intra-articular steroid, *QALY* quality-adjusted life-year

Results of the probabilistic sensitivity analysis for CRFA versus IAS at 6 and 12 months are depicted in Fig. 2 and Additional file 1: Figure S1. At a US$100,000 per QALY threshold, CRFA has an 86% probability of being cost-effective at 6 months and 95% at 12 months (Fig. 2).

## Discussion

Although the effectiveness and safety of CRFA in relieving pain and improving patient function in patients with symptomatic knee osteoarthritis has been clearly demonstrated in a randomized clinical trial [13, 14], to our knowledge, our study is the first to assess the cost-effectiveness of CRFA.

Using CRFA resulted in significant improvements in OKS versus IAS, which translated into ICERs below US$19,000 per QALY at 6 months and US$8000 per QALY at 12 months. The estimates reflect the sustained benefit of CRFA observed over 12 months, while the effect of IAS appears to wane. Analyses also included the cost of genicular nerve block, a diagnostic tool used to determine the potential responsiveness to ablation. Although nerve block was used on all trial subjects irrespective of therapy received (CRFA or IAS), it would have been inappropriate to include the costs of nerve block in the IAS arm in our analysis. As such, we assumed that only patients in the CRFA arm received nerve block and accrued the associated cost. Modifying the cost assumptions to reflect real-world clinical practice, including administration of IAS in-office without ultrasound guidance (versus outpatient facility using ultrasound) and genicular nerve block for CRFA patients given in-office (versus outpatient consultation), did not change the overall conclusion that CRFA is highly cost-effective versus IAS. All ICERs were well below the benchmark threshold of US$100,000 per QALY recommended in the US [28], representing the maximum amount that a decision-maker may be willing to pay for the health benefits provided by the treatment. Sensitivity analyses demonstrated the economic evaluation to be robust to variation in data inputs, and that there was a > 85% chance that CRFA is cost-effective compared with IAS at the US$100,000 per QALY threshold.

Utilizing data from the IAS-CRFA crossover group facilitated a comparison of early versus delayed CRFA, in which crossover patients accrued health benefits from CRFA post-IAS failure, but were attributed the cost of both IAS and CRFA treatment. This preliminary 12-month analysis suggested that immediate CRFA treatment may be cost-saving and offer greater QALY gains, compared with delaying CRFA until failure of IAS. The analysis included only those patients in the IAS arm who were dissatisfied and chose to switch; further evidence is required to robustly assess the cost-effectiveness of giving IAS first-line and offering CRFA only to those patients who fail treatment.

We are not aware of any previous economic evaluations on CRFA or any randomized trials comparing CRFA with interventions other than IAS, such as hyaluronic acid injections or other ablation techniques. Further research is required to examine how CRFA compares with other techniques from a clinical and an economic perspective.

Our analysis utilized trial data from a US multicenter study and took a US Medicare cost perspective. Although we are not aware of any evidence to suggest that the health benefits achieved by patients undergoing CRFA relative to IAS may be different for different geographic populations, differences in healthcare settings, clinical practice and associated costs mean that further research is required to assess cost-effectiveness in other countries.

Our findings should be interpreted considering several limitations. First, the 12-month CRFA-IAS analysis is limited by the large number of subjects who were dissatisfied with IAS after 6 months and who crossed over to receive CRFA, which resulted in only three subjects remaining in the IAS arm and having OKS data available at the 12-month follow up [13]. However, the treatment effect of IAS appeared to wane from Month 1 through to Month 6 post-treatment, whereas CRFA appears to have a durable effect out to 12 months. As such, more robust effectiveness data for IAS at 12 months (with larger sample sizes) would be unlikely to affect the overall conclusion that CRFA is cost-effective at a 12-month time horizon. Second, longer-term comparative clinical studies would allow estimates of cost-effectiveness beyond 12 months to be generated. Third, the trial and our economic analysis did not allow for repeat treatments, or switching to alternate treatments in the case of waning treatment effect. Although the CRFA treatment effect appears to be maintained to 12 months, CRFA may be repeated more frequently in practice, while the need for re-treatment beyond 12 months is unclear. Meta-analysis suggests that IAS can be effective for at least 16–24 weeks [9], while one study reported using a 3-monthly injection frequency over 2 years [8]. Further research may provide insight into the optimal timing of re-treatment with CRFA or IAS that can be incorporated into future cost-effectiveness analyses. Fourth, we excluded costs other than screening and treatment, consistent with the trial where all subjects were discharged to home with instructions for self-care. Follow-up costs, such as nurse follow-up and physiotherapy may be expected, however the improved effectiveness and durability of CRFA versus IAS would make it likely that these costs would be higher for IAS-treated patients. No significant differences in analgesia use between trial arms were observed, except for a significant reduction in non-opioid dose with CRFA at 6 months [14]. Overall, these cost exclusions would likely mean that the current analysis is conservative.

## Conclusions

In patients with symptomatic knee osteoarthritis, significant improvements in patient function with CRFA translate into health-related quality of life gains as early as 1 month, compared with conservative therapy with IAS injections. From the US Medicare perspective, CRFA is an efficacious and cost-effective treatment option for patients with osteoarthritis-related knee pain.

## Additional file

Additional file 1: Additional input data and results. Unit costs (inputs), tabulated parameters and values varied in sensitivity analyses (inputs), and scatter plots on cost-effectiveness plane for probabilistic sensitivity analysis (results). (DOCX 132 kb)

## Data Availability

In addition to the data inputs included in this published article and its supplementary information files, all data generated or analyzed during this study are available from the corresponding author on reasonable request.

## Abbreviations

CRFA: Cooled radiofrequency ablation
IAS: Intra-articular steroid
ICER: Incremental cost-effectiveness ratio
OKS: Oxford Knee Score
QALY: Quality-adjusted life-year

## Footnotes

1 FDA 510(k): K163461
