# Supplementary material for: Cooled radiofrequency ablation of the genicular nerves for chronic pain due to osteoarthritis of the knee: a cost-effectiveness analysis based on trial data
Source: BMC Musculoskelet Disord. 2019 Jun 26;20:302. doi: 10.1186/s12891-019-2681-2 (PMC6593544; doi:10.1186/s12891-019-2681-2)
Supplement: Supplementary file 1 — Additional input data and results. Unit costs (inputs), tabulated parameters and values varied in sensitivity analyses (inputs), and scatter plots on cost-effectiveness plane for probabilistic sensitivity analysis (results). (DOCX 132 kb) [file 12891_2019_2681_MOESM1_ESM.docx]

Additional file 1: Table S1. Unit costs (US$, 2017)

| **Cost parameter**  **Description (code)** | **Physician in-office payment allowable** | **Physician in-facility payment** | **Hospital outpatient payment** |
| --- | --- | --- | --- |
| **CRFA** |  |  |  |
| 1^st^ nerve: Destruction by neurolytic agent; other peripheral nerve neurolytic (CPT 64640) | - | 95.46 | 638.93 |
| 2^nd^ and 3^rd^ nerve: Destruction by neurolytic agent; other peripheral nerve neurolytic (CPT 64640-59) | - | 47.73 | 319.47 |
| Fluoroscopic guidance for needle placement (CPT 77002-26) | - | 28.71 | Bundled^†^ |
| Total hospital cost | $1,497.50 | | |
| **Genicular nerve block** |  |  |  |
| Injection, anesthetic agent; other peripheral nerve or branch (CPT 64450) | 81.47 | 46.66 | 507.19 |
| Fluoroscopic guidance for needle placement (CPT 77002) | 94.39 | - | - |
| Total hospital cost | $553.85 | | |
| Total in-office cost | $175.86 | | |
| **IAS** |  |  |  |
| Arthrocentesis, aspiration and/or injection, major joint or bursa (e.g., knee); with ultrasound guidance (CPT 20611) | - | 63.52 | 231.00 |
| Arthrocentesis, aspiration and/or injection, major joint or bursa (e.g., knee); without ultrasound guidance (CPT 20610) | 61.73 | - | - |
| Methylprednisolone acetate 40 mg (HCPCS J1030) ^‡^ | 6.00 | - | Bundled^†^ |
| Total hospital cost | $294.52 | | |
| Total in-office cost | $67.73 | | |

Abbreviations: CRFA, cooled radiofrequency ablation; IAS, intra-articular steroid.

†For procedures performed in the hospital outpatient setting, costs are bundled with the hospital outpatient payment.

‡Simplifying assumption; the majority (70%) of subjects in the trial received 40 mg methylprednisolone acetate.

Additional file 1: Table S2. Sensitivity analysis parameters

| **Variable** | **Current value** | **Lower value** | **Upper value** | **Distribution used in PSA** | **Source** |
| --- | --- | --- | --- | --- | --- |
| Genicular nerve block - Physician in-office payment; Injection, anesthetic agent; other peripheral nerve or branch (CPT 64450)^†^ | $81.47 | $73.32 | $89.62 | GAMMA | Centers for Medicare and Medicaid Services fee schedules [1] |
| Genicular nerve block - Physician in-hospital payment; Injection, anesthetic agent; other peripheral nerve or branch (CPT 64450)^†^ | $46.66 | $41.99 | $51.33 | GAMMA |  |
| Genicular nerve block - Hospital payment; Injection, anesthetic agent; other peripheral nerve or branch (CPT 64450)^†^ | $507.19 | $456.47 | $557.91 | GAMMA |  |
| Genicular nerve block - Physician in-office payment; Fluoroscopic guidance for needle placement (CPT 77002-26)^†^ | $94.39 | $84.95 | $103.83 | GAMMA |  |
| CRFA - Physician in-hospital payment; 1st nerve: Destruction by neurolytic agent; other peripheral nerve neurolytic (CPT 64640)^†^ | $95.46 | $85.91 | $105.01 | GAMMA |  |
| CRFA Hospital payment: 1st nerve - Destruction by neurolytic agent; other peripheral nerve neurolytic (CPT 64640)^†^ | $638.93 | $575.04 | $702.82 | GAMMA |  |
| CRFA - Physician in-hospital payment; 2nd/3rd nerve: Destruction by neurolytic agent; other peripheral nerve neurolytic (CPT 64640-59)^†^ | $47.73 | $42.96 | $52.50 | GAMMA |  |
| CRFA - Hospital payment; 2nd/3rd nerve: Destruction by neurolytic agent; other peripheral nerve neurolytic (CPT 64640-59)^†^ | $319.47 | $287.52 | $351.41 | GAMMA |  |
| CRFA - Physician in-hospital payment; Fluoroscopic guidance for needle placement (CPT 77002-26)^†^ | $28.71 | $25.84 | $31.58 | GAMMA |  |
| IAS - Physician in-office payment; Arthrocentesis, aspiration and/or injection, major joint or bursa (e.g., knee); without ultrasound guidance (CPT 20610)^†^ | $61.73 | $55.56 | $67.90 | GAMMA |  |
| IAS - Physician in-hospital payment; Arthrocentesis, aspiration and/or injection, major joint or bursa (egg, knee); with ultrasound guidance (CPT 20611)^†^ | $63.52 | $57.17 | $69.87 | GAMMA |  |
| IAS - Hospital payment; Arthrocentesis, aspiration and/or injection, major joint or bursa (e.g., knee); with ultrasound guidance (CPT 20611)^†^ | $231.00 | $207.90 | $254.10 | GAMMA |  |
| Steroids: Methylprednisolone acetate 40 mg (HCPCS J1030)^†^ | $6.004 | $5.40 | $6.60 | GAMMA |  |
| Mapping: OKS coefficient | 0.0224412 | 0.0223545 | 0.0225279 | NORMAL | Dakin et al [2] |
| Mapping: Constant | -0.0404485 | -0.0430593 | -0.0378377 | NORMAL |  |
| OKS - CRFA: Baseline | 16.7 | 15.7 | 17.7 | NORMAL | Davis et al [3]  Davis et al [4] |
| OKS - CRFA: Month 1 | 33.3 | 31.1 | 35.6 | NORMAL |  |
| OKS - CRFA: Month 3 | 34.6 | 32.6 | 36.6 | NORMAL |  |
| OKS - CRFA: Month 6 | 35.7 | 33.4 | 38.0 | NORMAL |  |
| OKS - CRFA: Month 12 | 34.3 | 31.3 | 37.3 | NORMAL |  |
| OKS - IAS: Baseline | 16.9 | 15.8 | 18.1 | NORMAL |  |
| OKS - IAS: Month 1 | 29.4 | 27.4 | 31.4 | NORMAL |  |
| OKS - IAS: Month 3 | 24.6 | 22.8 | 26.4 | NORMAL |  |
| OKS - IAS: Month 6 | 22.4 | 20.4 | 24.5 | NORMAL |  |
| OKS - IAS: Month 12 | 22.0 | 3.17 | 40.8 | NORMAL |  |
| OKS - IAS - CRFA crossover: Month 6 | 18.6 | 16.6 | 20.6 | NORMAL |  |
| OKS - IAS - CRFA crossover: Month 7 | 30.0 | 27.1 | 32.9 | NORMAL |  |
| OKS - IAS - CRFA crossover: Month 9 | 30.3 | 27.0 | 33.4 | NORMAL |  |
| OKS - IAS - CRFA crossover: Month 12 | 29.8 | 26.4 | 33.2 | NORMAL |  |

Abbreviations: CRFA, cooled radiofrequency ablation; IAS, intra-articular steroid; PSA, probabilistic sensitivity analysis; QALY, quality-adjusted life-year.

† Parameter varied by ±10%

Additional file 1: Figure S1. Cost-effectiveness plane for CRFA vs IAS. (A) 6-month time horizon. (B) 12-month time horizon

(A)


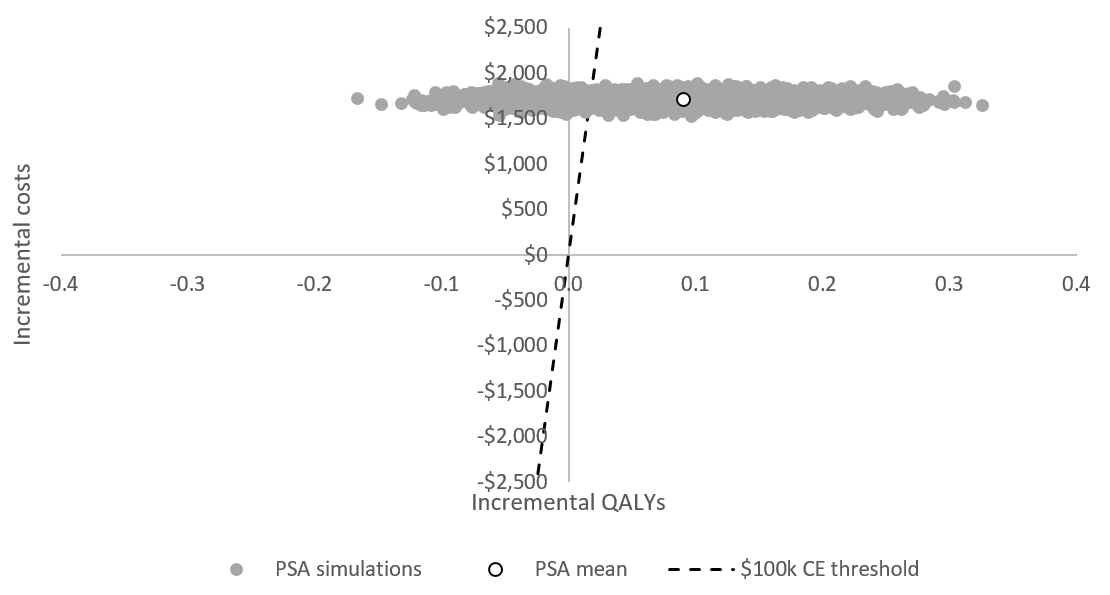


**(B)**


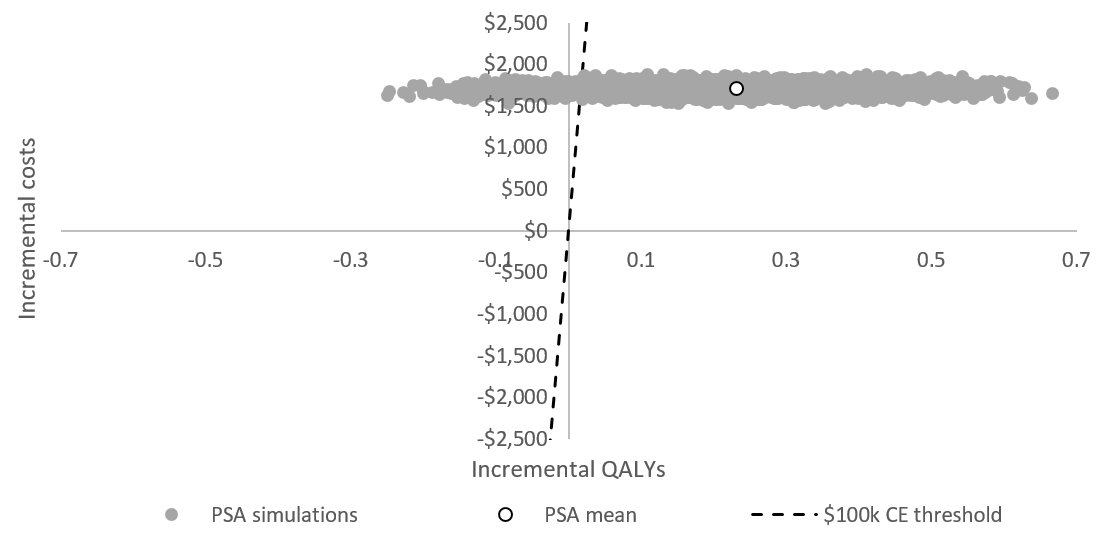


Abbreviations: CE, cost-effectiveness; CRFA, cooled radiofrequency ablation; IAS, intra-articular steroid; PSA, probabilistic sensitivity analysis; QALY, quality-adjusted life-year.

**REFERENCES**

1. Centers for Medicare & Medicaid Services. Medicare information for providers, partners and health care professionals. 2017. https://www.cms.gov/Medicare/Medicare.html. Accessed 2 January 2018.

2. Dakin H, Gray A, Murray D. Mapping analyses to estimate EQ-5D utilities and responses based on Oxford Knee Score. Spreadsheet algorithm for predicting EQ-5D utilities. Available at: http://www.herc.ox.ac.uk/downloads/mappingOKS. Qual Life Res. 2013;22(3):683-94.

3. Davis T, Loudermilk E, DePalma M, Hunter C, Lindley D, Patel N, Choi D, Soloman M, Gupta A, Desai M, Buvanendran A, Kapural L. Prospective, Multicenter, Randomized, Crossover Clinical Trial Comparing the Safety and Effectiveness of Cooled Radiofrequency Ablation With Corticosteroid Injection in the Management of Knee Pain From Osteoarthritis. Reg Anesth Pain Med. 2018;43(1):84-91.

4. Davis T, Loudermilk E, DePalma M, Hunter C, Lindley D, Patel N, Choi D, Soloman M, Gupta A, Desai M, Cook E, Kapural L. 12-month analgesia, and rescue, by cooled radiofrequency ablation treatment of osteoarthritic knee pain: Results from a prospective, multi-center, randomized, cross-over trial. Submitted for publication. 2018.
